# Alterations in Rev-ERBα/BMAL1 ratio and glycated hemoglobin in rotating shift workers: the EuRhythDia study

**DOI:** 10.1007/s00592-021-01676-z

**Published:** 2021-03-31

**Authors:** Stefano Rizza, Alessio Luzi, Maria Mavilio, Marta Ballanti, Arianna Massimi, Ottavia Porzio, Andrea Magrini, Juliane Hannemann, Rossella Menghini, Michael Lehrke, Bart Staels, Peter J. Grant, Rainer H. Boger, Nikolaus Marx, Massimo Federici

**Affiliations:** 1 Department of Systems Medicine, University of Rome Tor Vergata, Via Montpellier, 100133 Rome, Italy; 2 Department of Experimental Medicine, University of Rome Tor Vergata, Rome, Italy; 3 Department of Biomedicine and Prevention, University of Rome Tor Vergata, Rome, Italy; 4 Institute of Clinical Pharmacology and Toxicology, University Medical Center Hamburg-Eppendorf, Hamburg, Germany; 5 Department of Cardiology, University Medical Center Aachen, Aachen, Germany; 6 Univ. Lille, INSERM, CHU Lille, Institut Pasteur de Lille, U1011 - EGID, F-59000 Lille, France; 7 Leeds Institute of Cardiovascular and Metabolic Medicine, University of Leeds, Leeds, England

**Keywords:** Diabetes, Insulin resistance, Circadian, Obesity

## Abstract

**Objective:**

To detect premature gluco-metabolic defects among night shift workers with disturbances in circadian rhythms.

**Design and methods:**

We performed a hypothesis-generating, cross-sectional analysis of anthropometric, metabolic, lipid, and inflammation parameters, comparing active (a-NSW, *n* = 111) and former (f-NSW, *n* = 98) rotating night shift workers with diurnal workers (controls, *n* = 69). All participants were hospital nurses. We also evaluated the Pittsburgh Sleep Quality Index (PSQI) and assessed expression of transcription factors REV-ERBα and BMAL1 in peripheral blood mononuclear cells (PBMCs), as indicators of the molecular clock.

**Results:**

Both a-NSW and f-NSW participants had significantly higher glycated hemoglobin (HbA1c) and white blood cell counts (WBC) (*p* < 0.001 for both), PSQI global score (*p* = 0.001) and diastolic blood pressure levels (*p* = 0.024) compared with controls. Expression of REV-ERBα/BMAL1 RNA in PBMC was significantly higher in a-NSW (*p* = 0.05) than in f-NSW or control participants. Multivariate regression analysis showed that working status and PSQI were independent determinants of higher HbA1c levels (*p* < 0.001).

**Conclusions:**

We demonstrated that young, healthy night shift workers show subclinical abnormalities in HbA1c and changes in peripheral clock gene expression.

## Introduction

Night shift work (NSW) is highly prevalent in Western societies, with up to 20% of the European working population engaged in some type of shift work schedule [1], increasing to 45% in the healthcare sector [1]. Recently, epidemiological studies have suggested that rotating NSW is associated with a higher risk of developing type 2 diabetes (T2D) and cardiovascular diseases [2, 3]. Furthermore, even when diet is controlled, night shift workers exhibit poorer metabolic health than daytime workers [4]. Night shift workers, for example, exhibit higher plasma triglyceride levels [5], which is related to circadian system disruption; postprandial glucose and lipid tolerance to standard test meals are impaired after switching to NSW [6]. In addition, disrupted glucose metabolism and circadian misalignment during short sleep have been reported repeatedly [7, 8]. In the Nurse Health Study II, an increased risk of T2D was observed in participants with sleep difficulties doing rotating night shifts [9]. Moreover, in patients with overt diabetes, there is an association between glycemic control and sleep disturbances, while impaired melatonin secretion, insufficient duration and sleep quality have an adverse effect on insulin sensitivity and metabolic risk factors [10–12].

We conducted a hypothesis-generating, cross-sectional analysis of metabolic and circadian rhythm biomarkers in a group of hospital nurses recruited from the same working environment. Our results suggest that NSW in apparently healthy individuals is associated with subclinical defects that are typically manifested in people at risk of developing chronic metabolic disorders.

## Methods

### Participants

A group of volunteer hospital nurses was recruited at the Policlinico Tor Vergata University between 2012 and 2015 as part of the EuRhythDia project. The EuRhythDia consortium set out to investigate the disruption of the circadian clock in rotating night shift workers. A written consent was obtained from each patient after full explanation of the purpose and nature of all study procedures used. Participants received detailed information about the study protocol and, after providing written consent, underwent clinical examination and a standardized interview. Exclusion criteria included the presence of diabetes, liver disease, renal insufficiency, heart failure, coagulopathy or any other severe systemic disease. Subjects were also excluded if they had a history of any form of cancer or if they had positive blood tests for HIV, hepatitis B or hepatitis C or if they had taken melatonin supplements within four weeks before commencing the study.

In women, the study was initiated during the early follicular phase of the menstrual cycle.

Hospital workers were eligible if they had been working for a minimum of 2 years. We divided hospital nurses into three groups: [1] active rotating night shift workers (a-NSW, *n* = 111), working a shift schedule of from four to seven 12-h nights per month, followed by 2 days off; [2] former rotating night shift workers (f-NSW, *n* = 98), who had stopped night shift working for at least 2 years before inclusion in the study; and [3] daily workers (controls, *n* = 69): nurses who had never worked night shifts. In the overall population, body mass index (BMI) was calculated by dividing the weight (in kilograms) by the square of height (in meters). Three blood pressure measurements were obtained in the dominant arm in the sitting position and then averaged to calculate mean systolic- and diastolic-BPs. Current and former smokers were considered together as a single group and compared to never smokers.

### Clock genes analysis

After an overnight fast, blood samples and anthropometric parameters were obtained for controls and f-NSW between 8:00 and 9:00 AM whereas after 12 h of night shift while fasting conditions in a-NSW. About 20 ml whole blood was collected from each subject. Of these, about 8 mL was used for extraction of PMBC RNA and real-time quantitative RT-PCR analysis, as previously described [11]. Briefly, single-strand cDNA was synthesized, according to the Applied Biosystems (Foster City, CA) standard protocol, from 2 μg of total RNA sample using High-Capacity cDNA Archive Kit. Fifty nanograms of cDNA was amplified by real-time-PCR RNA expression of clock (BMAL1 and REV-ERBα), inflammatory (IL-1β, IF-γ) using an ABI PRISM 7500 System and normalized to 18S rRNA as an endogenous control [13].

### Sleep quality analysis

The Pittsburgh Sleep Quality Index (PSQI) is a validated and widely used scale to identify elements of sleep over the past 30 days and reflects the individual’s perception of sleep during shift and non-shift days [14].

This questionnaire identifies seven “components” of sleep routinely assessed clinically: sleep latency, sleep duration, habitual sleep efficiency, sleep disturbance, use of sleep medication, daytime dysfunction and subjective sleep quality. The sum of scores of the aforementioned seven components gives a global PSQI score that ranges from 0 to 21 points; a score of 5 or greater is associated with poor sleep quality and was therefore dichotomized as such in our analysis. The PSQI scores have been shown to have good test–retest reliability, with a correlation coefficient of 0.85 for the global score, and correlation coefficients ranging from 0.65 (medication use) to 0.84 (sleep latency) for the component scores [14, 15].

### Statistical analysis

Participants’ clinical characteristics were reported as means and standard deviations or as frequencies and percentages for continuous and categorical variables, respectively. Each continuous variable was checked for normality of distribution by the Kolmogorov–Smirnov test.

Univariate ANOVA was used to compare each of the reported variables in the a-NSW, f-NSW and control groups as described [16]. The Mann–Whitney test was used for variables with non-normal distribution.

The significance of difference between percentages in groups was evaluated by using the *χ*^2^ test. We used Spearman’s rho coefficient to calculate a nonparametric measure of rank correlation between PSQI score, glucose and glycated hemoglobin (HbA1c). We used linear regression analysis to explore independent associations between HbA1c entered into the model as the dependent variable and PSQI and working status as possible predictors controlled for age, sex and body mass index (BMI).

A *p*-value < 0.05 was considered statistically significant. All analyses were performed using SPSS for Windows software version 19.0 (IBM Corp., Armonk, NY, USA).

## Results

Anthropometric, clinical, metabolic and inflammatory characteristics of the study population are shown in Table 1. None of the participants had diabetes or other chronic metabolic or cardiovascular diseases. Participants in the a-NSW group (*n* = 111) were significantly younger (*p* < 0.001) than controls (*n* = 69) and f-NSW (*n* = 98), whereas the three groups did not differ significantly with respect to sex, lipid profile, presence of metabolic syndrome or numbers of smokers. Moreover, the groups were comparable regarding non-modifiable risk factors such as family history of diabetes. Mean HbA1c values were significantly higher in the a-NSW and f-NSW groups than in controls (*p* < 0.001), even after adjustment for BMI, age and sex (Table 1). Participants in a-NSW, compared with those in f-NSW and controls, had significantly higher BMI (*p* = 0.013) and creatinine (*p* = 0.038). No difference in systolic blood pressure (BP) was observed between groups (Table 1), whereas both a-NSW and f-NSW had significantly higher diastolic BP levels (*p* = 0.024), white blood cell (WBC) count (*p* < 0.01) and serum interleukin (IL)-1β levels (*p* = 0.014) compared with controls. Interestingly, diastolic, but not systolic, BP was significantly correlated with age (*r* = 0.129, *p* = 0.029). Mean values of metabolic parameters were similar, including fasting glucose and the degree of insulin resistance as measured using the homeostatic model assessment of insulin resistance (HOMA-IR). HbA1c was significantly correlated with WBC count (*r* = 0.153, *p* = 0.013) and with IL-1β (*r* = 0.138, *p* = 0.026) in PBMCs. Significant differences in the mean PSQI global score, a marker of sleep quality and circadian alignment, were observed, with a PSQI score > 5 in 20% of controls, in 51% of f-NSW workers and in 56% of a-NSW workers (*p* = 0.001) (Table 1). This difference persisted after adjusting for age, sex and BMI. Interestingly, the PSQI score was significantly correlated with HbA1c (*r* = 0.295, *p* < 0.001) but not with fasting glucose (*r* =  − 0.48, *p* = 0.420).

**Table 1:** Anthropometric, clinical, sleep, metabolic and inflammatory characteristics of the study population divided by working status: day workers (DW), active night shift workers (a-NSW) and former night shift workers (f-NSW)

Variables	DW (*n* = 69)	a-NSW (*n* = 111)	f-NSW (*n* = 98)	*p*
Age (years)	37.0 ± 7.2	36.1 ± 6.0	39.7 ± 7.1	< 0.001
BMI	23.7 ± 3.5	24.8 ± 3.7	25.7 ± 4.6	0.013
Gender (f/m)	39/25	74/37	72/26	0.236
Metabolic syndrome (y/n)	1/63	10/101	6/92	0.145
Smoker (y/n)	19/45	50/61	36/62	0.120
T2D first-degree relatives (y/n)	23/41	33/78	37/61	0.444
SBP (mmHg)	112.0 ± 12.0	114.0 ± 12.8	112.0 ± 13.3	0.450
DBP (mmHg)	71.0 ± 9.5	74.8 ± 9.3	73.6 ± 8.4	0.024
Fasting glucose (mg/dL)	89.7 ± 10.7	88.7 ± 8.1	89.9 ± 8.2	0.571
Fasting insulin (µU/mL)	10.4 ± 5.4	12.8 ± 14.3	12.0 ± 9.6	0.391
HOMA-IR	2.35 ± 1.37	2.63 ± 2.12	2.68 ± 2.24	0.566
HOMA-beta	149.2 ± 125.6	180.3 ± 170.9	174.8 ± 145.8	0.399
HbA1c (%)	5.12 ± 0.24	5.32 ± 0.32	5.33 ± 0.30	< 0.001*
Creatinine (mg/dL)	0.79 ± 0.15	0.82 ± 0.15	0.77 ± 0.13	0.038
Total cholesterol (mg/dL)	190.2 ± 36.5	189.7 ± 34.8	197.7 ± 37.0	0.231
HDL cholesterol (mg/dL)	58.5 ± 13.9	59.4 ± 16.6	56.4 ± 15.7	0.364
LDL cholesterol (mg/dL)	112.2 ± 33.2	110.4 ± 33.1	121.1 ± 33.0	0.047
Triglycerides (mg/dL)	97.4 ± 65.4	99.4 ± 67.7	101.3 ± 66.7	0.934
WBC count (× 10^3^/mL)	5.7 ± 1.2	6.9 ± 1.9	6.6 ± 1.7	< 0.001
CRP (mg/L)	1.4 ± 2.8	1.7 ± 3.0	2.1 ± 3.2	0.309
PSQI global score (score 1–4/5–21)	51/13	54/57	43/55	0.001*
*REV-ERBα* mRNA	1.298 ± 1.66	1.230 ± 0.626	1.218 ± 0.584	0.861
*BMAL1* mRNA	4.907 ± 6.693	4.170 ± 2.863	4.520 ± 2.804	0.506
*REV-ERBα*/*BMAL1*	0.310 ± 0.164	0.369 ± 0.210	0.318 ± 0.158	0.050
IFN-γ mRNA	0.74 ± 0.60	0.84 ± 0.87	0.99 ± 1.1	0.208
IL-1β mRNA	1.74 ± 3.91	8.94 ± 20.31	8.70 ± 20.40	0.014

*Adjusted for age, sex and BMI

**Fig. 1:**
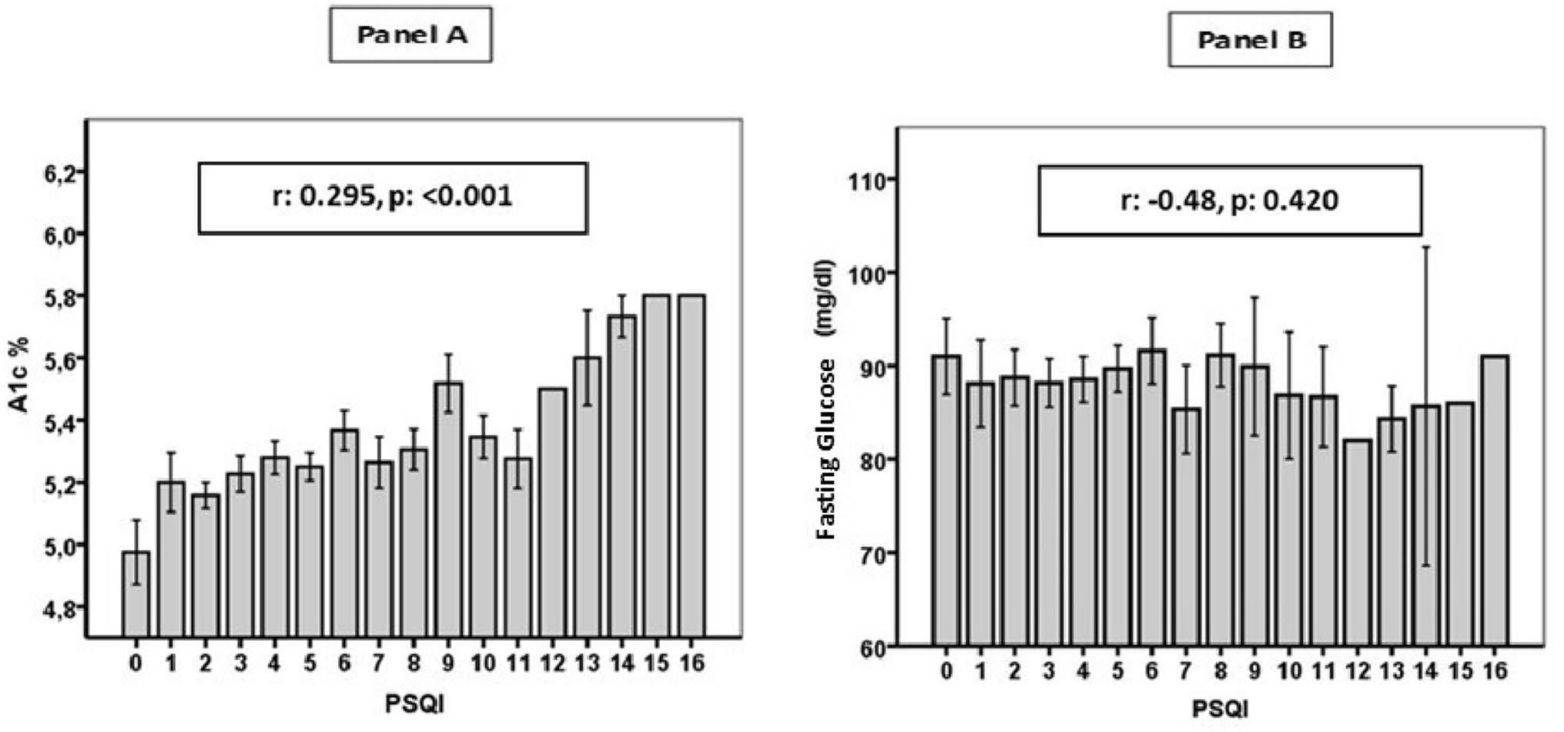
Correlation between Pittsburgh Sleep Quality Index (PSQI) and glycated hemoglobin (HbA1c, panel A), and PSQI and fasting glucose (panel B). Error bars indicate standard deviations

BMI, body mass index; T2D, type 2 diabetes; SBP, systolic blood pressure; DBP, diastolic blood pressure; HOMA-IR, homeostatic model assessment of insulin resistance; HbA1c, glycated hemoglobin; HDL, high-density lipoprotein; LDL, low-density lipoprotein; WBC, white blood cells; CRP, C-reactive protein; PSQI, Pittsburgh Sleep Quality Index questionnaire; IFN-γ, interferon-γ; IL-1β, interleukin-1β,

Next, we dichotomized the entire population in bad sleepers (BS, n = 110) and good sleepers (GS, *n* = 163) based on PSQI score cutoff > 5 (Table 2). BS were slightly older (*p* = 0.026) and more frequently female (*p* < 0.001). Interestingly, HbA1c level was the only metabolic variable significantly different between the groups, with a higher HbA1c in BS compared to GS (*p* = 0.025) even after adjustment for age, sex and BMI. Of note, the REV-ERBα/BMAL1 ratio was significantly lower in GS (*p* < 0.049). PSQI significantly correlated with HbA1c but not with fasting glucose (Fig. 1).

**Table 2:** Anthropometric, clinical, metabolic and inflammatory characteristics of the study population divided upon sleep quality

Variables	Good sleeper (*n* = 163)	Bad sleeper (*n* = 110)	*p*
Age (years)	36.9 ± 6.5	38.7 ± 6.3	0.026
BMI	24.9 ± 3.8	24.9 ± 4.4	0.992
Gender (f/m)	97/66	88/22	< 0.001
Metabolic Syndrome (y/n)	153/10	103/7	0.565
Smoker (y/n)	98/65	70/40	0.324
T2D first-degree relatives (y/n)	113/50	67/43	0.096
SBP (mmHg)	112.6 ± 12.9	113.1 ± 12.8	0.739
DBP (mmHg)	72.8 ± 8.7	74.5 ± 9.8	0.122
Fasting glucose (mg/dl)	89.4 ± 7.8	89.2 ± 10.2	0.866
Fasting Insulin (µU/ml)	11.6 ± 11.1	12.5 ± 11.2	0.515
HOMA-IR	2.43 ± 1.52	2.80 ± 2.57	0.141
HOMA-beta	156.9 ± 132.6	191.9 ± 187.8	0.088
HbA1c (%)	5.23 ± 0.29	5.33 ± 0.32	0.025 *
Creatinine (mg/dl)	0.79 ± 0.14	0.79 ± 0.14	0.864
Total cholesterol (mg/dl)	190.2 ± 34.0	196.5 ± 38.8	0.159
HDL cholesterol (mg/dl)	56.3 ± 15.5	60.8 ± 15.6	0.019
LDL cholesterol (mg/dl)	114.3 ± 31.5	115.2 ± 34.0	0.831
Triglycerides (mg/dl)	97.8 ± 67.0	102.2 ± 71.9	0.591
WBC (× 10^3^/ml)	7.04 ± 1.52	6.22 ± 1.50	0.103
C-reactive protein (mg/l)	1.43 ± 1.16	2.02 ± 2.1	0.548
REV-ERBα mRNA	1.27 ± 0.46	1.25 ± 1.0	0.944
BMAL1 mRNA	4.59 ± 4.40	5.30 ± 4.9	0.658
REV-ERBα/BMAL1	0.4 ± 0.1	0.3 ± 0.1	0.049
IFN-gamma	0.8 ± 0.8	0.9 ± 1.0	0.685
IL-1β	7.3 ± 20.0	6.77 ± 14.2	0.824

BMI, body mass index; T2D, type 2 diabetes; SBP, systolic blood pressure; DBP, diastolic blood pressure; HOMA-IR, homeostatic model assessment of insulin resistance; HbA1c, glycated hemoglobin; HDL, high-density lipoprotein; LDL, low-density lipoprotein; WBC, white blood cells; CRP, C-reactive protein; PSQI, Pittsburgh Sleep Quality Index questionnaire; IFN-γ, interferon-γ; IL-1β, interleuchin-1β

Then, we looked for markers of the positive and negative control of circadian clock in PBMCs. We observed no significant difference in leukocyte clock gene expression among the three groups (*p* = 0.506 for *BMAL1* and *p* = 0.861 for *REV-ERBα*). Although individual gene expression levels did not differ, we found that the *REV-ERBα*/*BMAL1* ratio was significantly higher in a-NSW than in f-NSW and control participants (*p* = 0.023). Of note, among all study covariates, *REV-ERBα*/*BMAL1* ratio expression was significantly correlated only with diastolic BP (*r* = 0.133, *p* = 0.024).

Multivariate regression analyses showed that both working status and PSQI were independent determinants of higher HbA1c levels, even after controlling for age, sex, BMI and a generic inflammation parameter such as WBC count. The model was highly significant (*F* = 9.7, *p* < 0.001) and explained 16.5% (adjusted R^2^) of the variance of HbA1c (Table 3).

**Table 3:** Multivariate linear regression with glycated hemoglobin (HbA1c) as dependent variable (*R*^2^: 0.165, *p* < 0.001)

Variable	Beta	95% CI for beta	*p*
Age (years)	0.008	0.002 to 0.013	0.005
Sex (male)	− 0.034	− 0.112 to 0.045	0.398
BMI	0.015	0.006 to 0.024	0.001
WBC count	0.021	− 0.001 to 0.042	0.063
PSQI	0.127	0.054 to 0.200	0.001
Working status (a-NSW)	0.053	0.006 to 0.101	0.028

BMI, body mass index; WBC, white blood cells; PSQI, Pittsburgh Sleep Quality Index questionnaire; a-NSW, active night shift workers

## Discussion

In this study, we observed that apparently healthy, young nurses doing rotating night shifts showed mild but significant increases in HbA1c levels compared with diurnal workers, independently of sex, age and BMI. HbA1c levels were also higher in f-NSW compared with diurnal workers. It is unclear whether the increase in HbA1c in f-NSW derived from some form of metabolic memory or whether NSW had changed lifestyle habits that were maintained after workers ceased NSW. However, our study shows that even relatively young healthy hospital workers, being or having been on night shift, in the absence of important cardiometabolic risk factors, show subclinical metabolic alterations that may be predictive of progression to more severe conditions such as diabetes.

The association of working status with HbA1c, but not with fasting glucose and HOMA-IR may be due to the increased glucose excursions during post-prandial states that might affect the hemoglobin glycation process. Consistently, it has been reported in humans that deregulated feeding and breakfast skipping may alter the function of the central and peripheral circadian clock and, as a result, impair glucose metabolism [17–19]. Similarly, circadian misalignment has been shown to increase inflammation in chronic shift workers [20]. Accordingly, since in study regression model WBC partly explained the increase of HbA1c, we tested the hypothesis that clock genes may be misaligned in PBMCs of a-NSW, which could contribute to increased inflammation [21].

Our data, showing an increase in expression of IL-1β RNA in PBMCs and high WBC count in a-NSW, and a significant correlation of HbA1c with leukocyte IL-1β RNA expression, suggest a potential common underlying mechanism for these changes and confirm the chronic low-grade inflammation is a recognized key feature associated with the risk of T2D development [22] and its complications [23].

Interestingly, we found that the *REV-ERBα*/*BMAL1* mRNA ratio was significantly higher in a-NSW compared with f-NSW and control participants. Actually, *REV-ERBα*/*BMAL1* mRNA ratio might be a potential indicator of desynchronization of the master clock caused by aberrant and prolonged artificial light exposure during night shifts [24–27]. However, even if the connection between the circadian clock and metabolism is well established [28], our results do not indicate whether the unbalanced *REV-ERBα*/*BMAL1* ratio derives from a disturbance of glucose metabolism or represents a marker of the proinflammatory state of circulating monocytes.

Finally, although sleep deprivation and population-based epidemiological studies have shown that disrupted sleep is a risk factor for hypertension, we observed a significant increase only in diastolic, not systolic, BP in a-NSW compared with the other two groups. However, considering the young mean age of our study population and the significant correlation of age with diastolic BP, our results seem to be consistent with emerging data indicating that the adverse cardiovascular consequences of disrupted sleep may begin during early adulthood [29, 30].

Our work has limitations, including its cross-sectional design and lack of prospective analysis. Moreover, the blood sampling time point after a night shift for a-NSWs may have confounded some variables that vary across the day, including BP, fasting glucose, WBC count, gene expression of *REV-ERBα*/*BMAL1*, but not HbA1c level. Furthermore, the *REV-ERBα*/*BMAL1* ratio as biomarker of circadian rhythm should be assessed in other cohorts with samples that are available from several time points during the 24 h.

Future studies are warranted to understand how long it may take to increase the glycemic-inflammatory burden in night shift workers and to design appropriate strategies to dampen this effect.

## Abbreviations

NSW: Night shift work
PSQI: Pittsburgh Sleep Quality Index
PBMCs: Peripheral blood mononuclear cells
HbA1c: Glycated hemoglobin
